# Lung ultrasound in diagnosis of acute heart failure: A protocol for systematic review and meta-analysis

**DOI:** 10.1097/MD.0000000000032257

**Published:** 2022-12-09

**Authors:** Hao Chen, Yang Chen, Shidong Liu, Guangzu Liu, Hongxu Liu, Bing Song

**Affiliations:** a The First Clinical Medical College of Lanzhou University, Lanzhou University, Lanzhou, Gansu Province, China; b Department of Cardiovascular Surgery, First Hospital of Lanzhou University, Lanzhou, Gansu Province, China.

**Keywords:** diagnosis, heart failure, lung ultrasound, meta-analysis

## Abstract

Lung ultrasound has been used more and more in clinical diagnosis of acute heart failure (AHF) in recent years. The purpose of this paper is to evaluate the diagnostic value of lung ultrasound in AHF by meta-analysis.

**Methods:**

PubMed, Cochrane Library, Web of Science, and Embase were searched. The time limit for retrieval is from the establishment to October 2022. According to the criteria, the literatures were screened and the relevant data was extracted. Efficacy of lung ultrasound in AHF was evaluated using Stata Version 16.0, (Stata Corp, College Station, TX).

**Results:**

This study will be submitted to a peer-reviewed journal for publication.

**Conclusion:**

This study conducted a systematic review of relevant studies, which aims to systematically evaluate the diagnostic value of lung ultrasound in AHF.

## 1. Introduction

Acute heart failure (AHF) is caused by left ventricular diastolic or systolic dysfunction leading to an increase in preload and postload, further leading to pulmonary congestion, fluid retention and redistribution, and finally to organ dysfunction due to hypoperfusion, which endangers the patient’s life.^[1,2]^ AHF has become an increasingly serious public health problem in recent years as the population over the age of 65 continues to grow.^[3]^ AHF is also a common cause of emergency admissions, and the 2016 European Society of Cardiology Guidelines for the Diagnosis and Treatment of Acute and Chronic Heart Failure state that echocardiography remains the most useful and widely used diagnostic method.^[4]^ However, other traditional diagnostic methods, such as medical history and physical examination, electrocardiogram, chest X-ray, serum N-terminal precursor brain natriuretic peptide, lack specificity or sensitivity, and have high requirements on equipment, time and other factors, or are limited by radiation to the applicable population, so they cannot make a rapid and accurate diagnosis of AHF. So far, there are still many problems in the efficient diagnosis of AHF.^[5]^

Lung ultrasound was first proposed in the 1990s and has been used in a number of clinical critical cases. Studies have shown that lung fluid accumulation in patients with AHF leads to a decrease in the ratio of air to fluid in the lung, resulting in an increase in acoustic impedance, which is different from surrounding tissues. Sound waves will generate multiple reflections. Therefore, there are multiple acoustic interfaces between fluid and alveoli, comet tail signs (B-lines) can be seen on ultrasound scans of the lungs. Through continuous development and application, lung ultrasound has been proved to be of value in the diagnosis of AHF in recent years, providing clinicians with a simple, rapid, mobile and noninvasive tool.^[6–8]^ Based on the above advantages, lung ultrasound is more and more used in the diagnosis of AHF. At present, there are a variety of zonal lung ultrasound scanning methods for the diagnosis of AHF, mainly including 4-point method, 6-point method, 8-point method and 28-point method.^[9]^

This paper aims to systematically evaluate the diagnostic value of lung ultrasound in AHF through existing studies and obtain reasonable comprehensive data, in order to provide a reference for clinical practice.

## 2. Methods and analysis

### 2.1. Search strategy

PubMed, Cochrane Library, Web of Science and Embase were searched. The time limit for retrieval is from the establishment to October 2022. The keywords include “Cardiac Failure,” “Heart failure,” “Diagnostic Ultrasound,” “Ultrasonography,” and “Lung.” Further, we manually searched for relevant studies in a reference list of potentially eligible publications.

### 2.2. Inclusion and exclusion criteria

All qualifying studies should meet the following criteria: adults with symptoms of AHF were included; the types of studies included were cohort studies; the gold standard for inclusion was the clinical diagnosis of heart failure by cardiologists or experienced physicians combined with history and relevant clinical examination; the language of the included literature was English; there is no restriction on the type of ultrasound instrument and the method of zonal lung ultrasound scanning, and the location of pulmonary ultrasound examination is not limited to the emergency department. Studies were excluded if they met the following criteria: duplicate publications; conference reports, reviews, meta-analyses, editorials, letters, and case reports were excluded; studies with a sample size of <20 were excluded; excluded studies where data could not be extracted or were incomplete.

### 2.3. Data extraction and analysis

Two researchers independently extracted relevant data according to the predesigned data extraction table, and then cross-checked the results. In case of disagreement, discussion or decision by a third person will be carried out. The main extraction contents included: first author, publication year, study type, ultrasonic instrument, probe type, ultrasonic zonal scanning method, true positive value (TP), false positive value (FP), false negative value (FN), true negative value (TN), sample size, etc.

### 2.4. Quality assessment

Quadas-2 tool (Quality Assessment of Diagnostic Accuracy Studies - 2) was used for quality evaluation of the included studies, and RevMan5.3 software (Version 5.3, The Cochrane Collaboration, 2020.) was used to create and generate the figure of risk of bias.

### 2.5. Statistical analysis and assessment of heterogeneity

Statistical analysis was performed using Stata 16.0 software, and Bivariate mixed effects model was applied. Firstly, the meta-disc 1.4 software (Version 1.4;the Ramón y Cajal Hospital in Madrid,Spain) was used to evaluate the threshold effect by Spearman correlation analysis. If there is no threshold effect among studies, evaluation indexes can be directly combined. The sensitivity, specificity, positive likelihood ratio, negative likelihood ratio and diagnostic odds ratio were calculated. Forest plot of sensitivity and specificity were also drawn. summary receiver operating characteristic curve can also be fitted. On the contrary, if there is threshold effect between studies, summary receiver operating characteristic curve should be fitted directly. Cochran *Q* test and *I*^2^ value were used to analyze heterogeneity. Cochran *Q* test *P* < .05 and *I*^2^ > 50% were considered significant heterogeneity. Meta-regression was performed to further explore the source of heterogeneity. Using Fagan plot to display the posterior probability.

### 2.6. Publication bias

Deeks funnel plot asymmetry test was used to determine whether there was publication bias. *P* < .05 was considered statistically significant.

## 3. Discussion

AHF has an acute onset, and the mortality rate has not decreased significantly over the years. With the aging trend of the population, the number of patients will continue to expand. Therefore, early diagnosis has become an important factor for rapid development of appropriate treatment and reduction of heart failure mortality. In recent years, lung ultrasound has been increasingly used in clinical practice. It can be used to diagnose pulmonary edema, evaluate the degree of pulmonary edema in AHF, and has certain value in the diagnosis of pulmonary consolidation, pulmonary embolism, pneumothorax, etc.^[10,11]^ This study conducted a systematic review of relevant studies, which aims to systematically evaluate the diagnostic value of lung ultrasound in AHF.

## Author contributions

Hao Chen and Yang Chen designed the study and were the main coordinators of the study. Hao Chen was the principal investigator and guarantor. Hao Chen and Shidong Liu conducted the study. Yang Chen, Hongxu Liu and Guangzu Liu gave statistical and epidemiological support. Hao Chen wrote the article with the support of Bing Song, Shidong Liu.

**Conceptualization:** Yang Chen.

**Data curation:** Hongxu Liu.

**Formal analysis:** Guangzu Liu.

**Funding acquisition:** Hongxu Liu, Bing Song.

**Investigation:** Bing Song.

**Methodology:** Shidong Liu.

**Resources:** Bing Song.

**Software:** Yang Chen, Shidong Liu.

**Writing – original draft:** Hao Chen.

**Writing – review & editing:** Hao Chen.
